# Dynamics of Chytridiomycosis during the Breeding Season in an Australian Alpine Amphibian

**DOI:** 10.1371/journal.pone.0143629

**Published:** 2015-12-02

**Authors:** Laura A. Brannelly, David A. Hunter, Daniel Lenger, Ben C. Scheele, Lee F. Skerratt, Lee Berger

**Affiliations:** 1 One Health Research Group, College of Public Health, Medical and Veterinary Sciences, James Cook University, Townsville, Queensland, Australia; 2 Ecosystems and Threatened Species, South West Region, Office of Environment and Heritage, NSW Department of Premier and Cabinet, Albury, New South Wales, Australia; Vanderbilt University School of Medicine, UNITED STATES

## Abstract

Understanding disease dynamics during the breeding season of declining amphibian species will improve our understanding of how remnant populations persist with endemic infection, and will assist the development of management techniques to protect disease-threatened species from extinction. We monitored the endangered *Litoria verreauxii alpina* (alpine treefrog) during the breeding season through capture-mark-recapture (CMR) studies in which we investigated the dynamics of chytridiomycosis in relation to population size in two populations. We found that infection prevalence and intensity increased throughout the breeding season in both populations, but infection prevalence and intensity was higher (3.49 and 2.02 times higher prevalence and intensity, respectively) at the site that had a 90-fold higher population density. This suggests that *Bd* transmission is density-dependent. Weekly survival probability was related to disease state, with heavily infected animals having the lowest survival. There was low recovery from infection, especially when animals were heavily infected with *Bd*. Sympatric amphibian species are likely to be reservoir hosts for the disease and can play an important role in the disease ecology of *Bd*. Although we found 0% prevalence in crayfish (*Cherax destructor*), we found that a sympatric amphibian (*Crinia signifera*) maintained 100% infection prevalence at a high intensity throughout the season. Our results demonstrate the importance of including infection intensity into CMR disease analysis in order to fully understand the implications of disease on the amphibian community. We recommend a combined management approach to promote lower population densities and ensure consistent progeny survival. The most effective management strategy to safeguard the persistence of this susceptible species might be to increase habitat area while maintaining a similar sized suitable breeding zone and to increase water flow and area to reduce drought.

## Introduction

The amphibian disease chytridiomycosis (caused by the fungal pathogen *Batrachochytrium dendrobatidis*, *Bd*) is a major cause of amphibian declines globally and has been called the most devastating threat from disease to biodiversity [1]. In-depth ecological studies are important for determining disease impact in the wild because many factors can affect disease dynamics. Breeding habitat plays an important role in the prevalence of *Bd* infection, with higher prevalence more often associated with permanent water bodies [2,3]. *Bd* is known to exhibit density-dependent disease transmission [4–6]. *Bd* infection tends to peak seasonally [7–11], and the peak is often attributed to optimal temperature conditions for *Bd*. However, other factors might play a role [12]. Aggregate breeders often experience dramatic increases in infection prevalence during their short breeding season [13], likely due to both increased density of animals and increased frequency of contact due to breeding behaviour. Intensive population monitoring throughout the breeding season can shed light on the ecological impact of *Bd* on declining species and can inform management decisions.

Capture-mark-recapture (CMR) studies are an effective ecological tool to determine effects of disease on populations and individuals in the wild and guide management decisions [14]. The in-depth analysis of CMR data allows for greater understanding of how survival and recapture probability are directly affected by disease and the probability of an animal gaining and recovering from infection [15]. CMR studies in amphibian populations with prevalent *Bd* infection have uncovered factors that influence disease dynamics including a dependence on disease prevalence, infection intensity, temperature, population density, and reservoir hosts [5,14,15]. Infection intensity is known to play an important role in disease dynamics, yet CMR studies often do not separate animals with heavy versus light infection [5,16]. It is important, particularly for pathogens with a strong relationship between infection burden and disease impact, to conduct CMR analyses that include infection load in order to obtain a true understanding of the disease dynamics [5].

Here, we conducted a CMR study to monitor the effects of prevalence and intensity of *Bd* infection in the endangered alpine treefrog, *Litoria verreauxii alpina*, with the purpose of better understanding the ecology of chytridiomycosis during the breeding season. Our most important aim was to identify opportunities for intervention that would promote the recovery of this and similar species. *Litoria v*. *alpina* is an aggregate breeder and native to the upland regions of the Australian Alps above 1200 m. Once widespread, *L*. *v*. *alpina*’s distribution has declined by over 80% of its former range since the 1980s [17] due primarily to chytridiomycosis [18]. Despite major declines, some populations persist in the presence of the pathogen [17,18]. While adults are known to have high mortality due to the disease, tadpoles and juveniles do not; therefore, high recruitment plays an important role in population persistence [19]. Although this species is endangered, populations have not been intensively monitored for chytridiomycosis during the breeding season when animals are thought to gain infection.

A second aim to this study was to screen for potential *Bd* reservoir species. Disease reservoirs can play an important role in the disease dynamics of co-occurring susceptible species [5]. A possible amphibian reservoir species present in the Australian Alps is the widespread and abundant common eastern froglet, *Crinia signifera* [18,19]. Recent research of non-amphibian hosts of *Bd* identified the North American crayfish, *Procambarus clarkii* [20,21]. While *P*. *clarkii* is not present in Australia, the commercially farmed and invasive crayfish species *Cherax destructor* is widespread throughout Australia [22] and might be involved in the dynamics of chytridiomycosis.

## Materials and Methods

### Study site

We conducted an intensive 10-week CMR study of two populations of *L*. *v*. *alpina* during their 12-week breeding season (see S1 File) [23]. Study Site 1: Oglivies Dam is a 0.17 hectares low elevation (1382m) site (S1 File) [23]. Sampling occurred between 1-Sept-2013 through 6-Nov-2013 (weeks 2–11 of breeding). Study Site 2: Sponar’s Creek (S1 File) [23] is a 1.8 hectares high elevation (1515m) site. Sampling occurred between 25-Sept-2013 and 25-Nov-2013 (weeks 1–10 of breeding) (S1 Fig). Air and water temperatures were recorded every two hours at each site using iButtons (S2 Fig).

### Field survey

Animals were captured during one to three nights each week over 10 weeks, resulting in the capture of up to 50 new animals on each survey night (S1 Table). Animals were captured with a new, clean, gloved hand and kept individually in a new plastic zip bag. Animals were set aside while we completed our collection and then processed and returned to the site of capture each night. Animals found in amplexus were held together in one bag. Animals were photographed (for individual identification, see S1 File), swabbed for *Bd* (see below), weighed to the nearest 0.01g and snout to venter length (SVL) was measured to the nearest 0.02mm. Waders and boots were dried between sites to prevent the spread of *Bd*.

### Testing for *Bd*

We tested for *Bd* infection by using skin swabs and a qPCR assay [24]. The swabbing protocol was standardized by performing 45 strokes on the venter and limbs with a sterile rayon-tipped swab (MW-113, Medical Wire & Equipment, Wiltshire, United Kingdom). Genomic DNA was extracted from the swabs using the Prepman Ultra (Applied Biosystems®, Life Technologies Pty Ltd, Carlsbad, California, USA) and a bead beater to break the fungal cell walls for two minutes, and then the extract was diluted 3:47 in PCR water. Extracted DNA was then analysed using quantitative real time PCR following Boyle *et al*. [24]. We conducted the analysis in singlicate to maximise both cost efficiency and test accuracy [25,26] including a positive and negative control and a series of dilution standards (to estimate infection load in zoospore equivalents, ZE).

To test for inhibition of the swab DNA, a subset of 20 samples was haphazardly selected and an internal positive control (VIC^TM^ IPC, Applied Biosystems®, Life Technologies Pty Ltd, Carlsbad, California, USA) was added to the qPCR reaction. No inhibition was detected in those samples. Because many samples returned high (>1000ZE) infection loads and prevalence was high, we concluded that inhibition due to high zoospore loads was unimportant. We prioritized resources to increase sample size rather than including IPC’s in every reaction.

### Reservoir hosts

Common eastern froglets (*Crinia signifera)* were collected at both sites (n = 93) between 21-Sept-2013 and 21-Nov-2013 (S2 Table). Animals were collected and individually stored in a clean plastic bag, and swabbed for *Bd* using the same protocol as for *L*. *v*. *alpina* and then released.

The western blue claw yabbie, *Cherax destructor*, was present at both sites where *L*. *v*. *alpina* were sampled, but it proved difficult to capture. Instead, crayfish were collected from a nearby site that supported populations of *L*. *v*. *alpina* and *C*. *signifera*: Kiandra (S1 Fig; S2 Table) (Lat. -35.867, Long. 148.498: Elevation 1358m). Frogs at this site have similarly high *Bd* prevalence [19]. Animals were collected with baited (Fancy Feast^TM^, Nestlé Purina PetCare, St, Louis, Missouri, USA) minnow traps between 1-Oct-2013 and 25-Nov-2013. Traps were left open for 2–5 days before collection. Animals were collected individually from the traps with inverted plastic zip bags, and euthanized by freezing for at least two hours (n = 94).

The gastrointestinal (GI) tract was tested for *Bd* presence following Brannelly *et al*. [20]. Because faecal matter inside of the GI tract causes PCR inhibition, it was removed with a sterile swab. The inside of the GI tract was then swabbed using 30 strokes with a new sterile MW113 swab, and the GI tract was swabbed again with a second swab as a backup. We followed the same extraction and qPCR protocol as for the frogs except that we diluted DNA extraction samples 1:10. We included an IPC in each sample to test for inhibition. Samples were analysed in singlicate, but inhibited samples were reanalyzed in triplicate. When all samples returned negative results for *Bd*, we selected a subset of 24 backup swabs and re-extracted the DNA using Qiagen DNeasy Blood and Tissue Kit using a final elution volume of 200μl. The samples were then analyzed following the above stated qPCR protocol, but the DNA sample was undiluted. No evidence of inhibition was observed.

### Statistical analysis

Survival, recapture probability, and disease state were examined using the statistical software M-SURGE [27]. The Conditional Arnason–Schwarz model was used. M-SURGE is a program designed specifically for multi-state CMR studies and can be used to analyse low recapture rates. We determined survival (S), recapture (r) and state change (Ψ) probabilities. The independent variables tested were site (s: Oglivies Dam and Sponar’s Creek), time in weeks (t), current *Bd* state (f), and *Bd* state of previous capture (to). The two *Bd* states were *Bd* positive and *Bd* negative. Only data from week 3 through week 10 of the breeding season were included, because both sites were sampled for those eight weeks. We measured goodness of fit as “ĉ” with the program U-CARE, and ĉ = 0.707. Below a value of “1”, ĉ represents under distribution of the data; therefore, ĉ not adjusted, and ĉ = 1 was used in analysis. Because females were never recaptured, they were excluded from the analyses in M-SURGE. While typically only two disease states are investigated [15,28–30] (*Bd* negative and *Bd* positive) as above, we decided to further explore the effect of infection intensity. To explore infection intensity, we analysed the data using a three-disease-state model in which the three states were *Bd* negative, low infection (<350 zoospore equivalents, ZE) and high infection (>350ZE). The goodness of fit was ĉ = 0.661, and it was not adjusted.

Population size for each population was analysed using a Program MARK POPAN model. Variables explored were (t) time in weeks, (Φ) survival probability, (p) recapture probability, and (Pent) probability of entry into the population. Because males were more conspicuous than females and tend to be present at the breeding site for an extended period, the “super-population size” calculated through POPAN refers to all the males that came to breed within the 2013 breeding season. Population density was estimated as the super-population size of males divided by site area.

Model selection was based on Akaike’s information criterion (AIC), with the best fitting model indicated by the lowest AIC value. The seven best fitting models were chosen for analysis. We did not perform model averaging.

Infection intensity over the breeding season was assessed using ANCOVA in SPSS (v21), and individuals are covariates. The data were transformed Log_10_(N+1) for a normal distribution, and only positive animals were included in the analysis. Infection prevalence was assessed using a logistic regression in which change in infection over time was compared between populations. Prevalence between the two sites within specific weeks was compared using Pearson’s Chi-Squared test in SPSS (v21), and odds ratios were calculated in Microsoft Excel. Because female capture rates were low, infection intensity and prevalence analyses only included males. To compare overall infection status of males and females between the two sites, ANCOVAs in SPSS (v21) were used, in which sex, site, week, site*sex and week*sex were the factors analysed, and individual was a covariate.

Body condition defined as mass/SVL was analysed using ANCOVAs in SPSS (v21). Mass/SVL is an appropriate measure for body condition in this species because it is highly correlative (Female: Pearson’s correlation = 0.817, p < 0.01; Male: Pearson’s correlation = 0.669, p < 0.01) and linear (Linear Regression ANOVA: Female: Mass = -5.819 + 0.272(SVL) p < 0.01; Male: Mass = -1.598 + 0.121(SVL), p < 0.01) [31]. We compared site and time, and individuals were covariates. Effect size was determined using the averaged Cohen’s *d* statistic across time and was calculated in Microsoft Excel. The effect of infection intensity on body condition was correlated using linear regression analysis in SPSS (v21), and time was not included.

### Animal ethics and permits

This study was carried out in strict accordance with the recommendations of Animal Ethics. The protocol was approved by the Animal Ethics Committee at James Cook University (Application A1880). All necessary permits obtained for the described study complied with all relevant regulations. Amphibian and crayfish collection permits were issued by NPWS Wildlife Licencing and Management.

## Results

### Population density

The best supported model to estimate population abundance of males over the course of the breeding season was Φ(.)p(t)pent(t) for both sites (Oglivies Dam; AIC = 439.318, parameters = 16: Sponar’s Creek; AIC = 816.459, parameters = 17) (Table 1). The outcomes explored were Φ = survival probability, p = recapture probability and pent = probability of entry into the population. The variable that could affect each outcome was t = time in weeks, or (.) indicating that time was not an important variable in predicting the outcome.

**Table 1:** AIC ranking of best-fit POPAN models. Model results are for the two sites: Sponar’s Creek and Oglivies Dam.

Model Variables*	Parameters	Deviance	AIC	Δ AIC
*Sponar’s Creek*				
Φ(.)p(t)Pent(t)	17	0	816.459	0
Φ(t)p(.)Pent(t)	16	0	826.993	10.534
Φ(t)p(t)Pent(t)	24	0	829.007	12.548
Φ(.)p(.)Pent(t)	8	0	830.658	14.199
Φ(.)p(.)Pent(.)	3	21818.006	23201.514	22385.055
*Oglivies Dam*				
Φ(.)p(t)Pent(t)	16	0	439.318	0
Φ(t)p(t)Pent(t)	23	0	449.208	9.89
Φ(t)p(.)Pent(t)	18	0	449.832	10.514
Φ(.)p(.)Pent(t)	9	0	456.482	17.164
Φ(.)p(.)Pent(.)	3	67125.025	70049.5921	69610.2741

* Outcome probabilities determined are (Φ) survival probability, (p) recapture probability, and (Pent) probability of entry into the population. These outcome probabilities are determined by the variable (t) time in weeks, or no variable (.).

The population estimate for Oglivies Dam (0.17 hectares) was 2725 males (95% CI, 1712–4505); and the estimated population density was 16,031 males per hectare (95% CI, 10,070–25,498) (Table 2). Sponar’s Creek (1.8 hectares) population size was smaller with 319 males (95% CI, 277–381); and the estimated population density was 177 males per hectare (95% CI, 154–212) (Table 2). The population density of *L*. *v*. *alpina* at Oglivies Dam was on average 90.53 times higher than at Sponar’s Creek.

**Table 2:** Recaptured animals are each site. Number of *Litoria verreauxii alpina* that were recaptured at the different sites (Sponar’s Creek, 1.8 hectares in area: Oglivies Dam, 0.17 hectares in area) and how many times the individual was recaptured over the course of the breeding season. Population size is the super-population estimate for males.

Number of recaptures	Oglivies Dam	Sponar's Creek
0	201	139
1	40	50
2		17
3		11
5		1
*Total captured*	241	218
*Population size*	2725	319

### Recaptures

A total of 459 animals were captured at both sites over the course of the breeding season. At Oglivies Dam, 241 animals were captured, 18 were female, and 16.60% of males were recaptured (Table 2). At Sponar’s Creek, 218 animals were captured, 21 were female, and 36.24% of males were recaptured (Table 2).

### Disease dynamics

**Fig 1:**
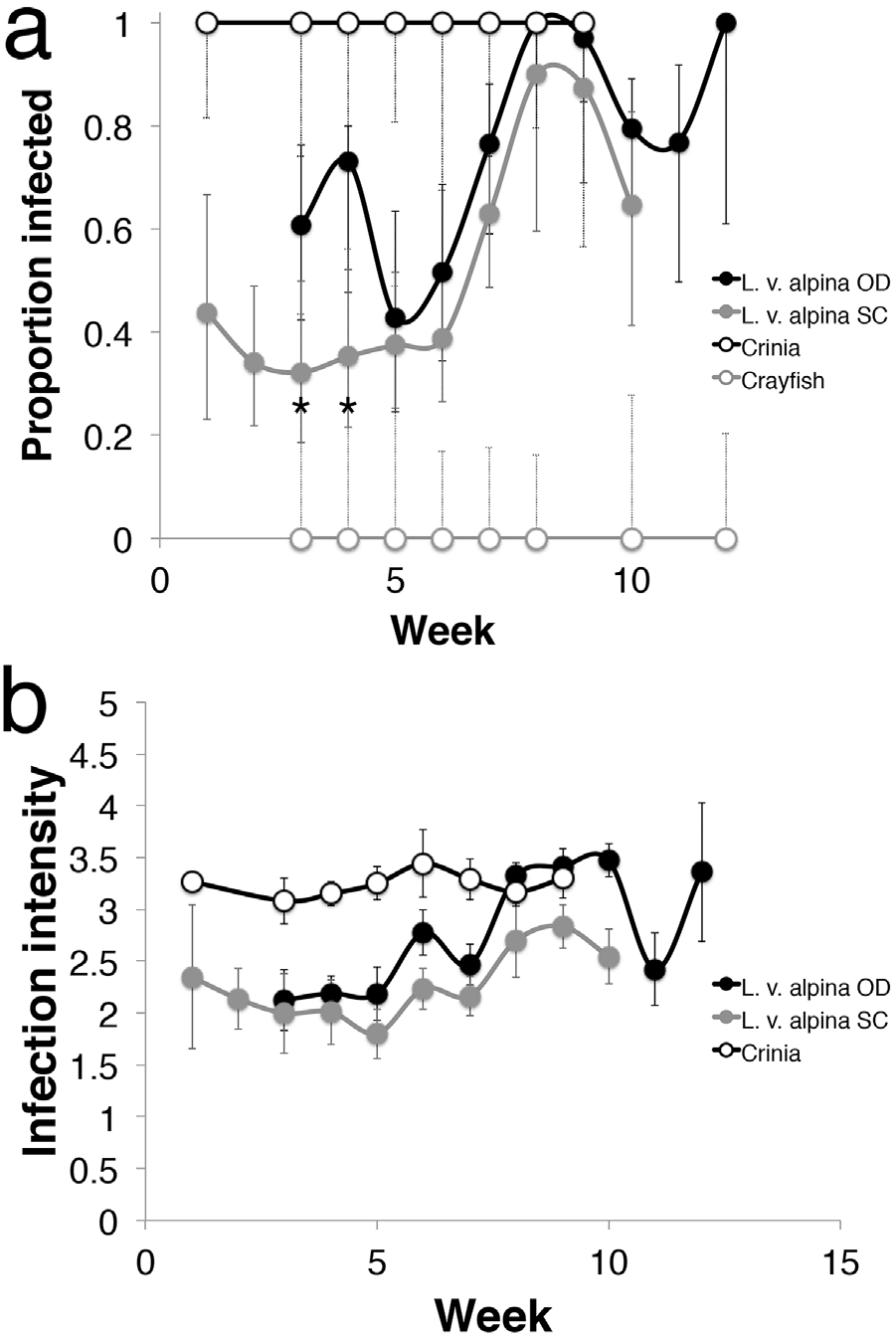
Infection prevalence and intensity across sites for *Litoria verreauxii alpina*, *Crinia signifera* and *Cherax destructor*. (a) Prevalence of infection. Error bars indicate 95% confidence intervals. (*) indicates time-points where prevalence is significantly different at each site for *Litoria verreauxii alpina*, using Pearson’s Chi-Squared test. (b) Infection intensity transformed to log_10_ scale. Error bars indicate standard error. Sites are Oglives Dam (OD) and Sponar’s Creek (SC).

Between recaptures, the disease state detected for some animals often changed (Table 3). The most common disease state change was from *Bd* negative to low infection intensity at Sponar’s Creek, and from low infection intensity to high infection intensity at Oglivies Dam. It was less common for an animal at either site to reduce or clear infection (Table 3). Therefore, infection prevalence increased each week throughout the course of the breeding season at both sites (Logistic Regression: Exp(B) = 1.313, p<0.001) and also differed significantly between sites (Logistic Regression: Exp(B) = 0.319, p<0.001). Oglivies Dam had higher infection prevalence than Sponar’s Creek throughout the breeding season (Odds ratio = 3.50). Oglivies Dam infection prevalence was greatest at weeks 3 (Chi-Squared: χ^2^ = 4.800, p = 0.028; Odds ratio = 3.25) and 4 (Chi-Squared: χ^2^ = 8.422, p = 0.004; Odds ratio = 4.98) (Fig 1A).

**Table 3:** Disease state change over the course of the study. The proportion of recaptured individuals that changed disease state over the course of multiple recaptures between the two sites: Oglivies Dam and Sponar’s Creek. This table represents the data collected from the CMR study.

Recaptures	Oglivies Dam	Sponar’s Creek
Stay Zero	0.025	0.266
Stay Low	0.075	0.063
Stay High	0.250	0.025
Zero to Low	0.125	0.266
Zero to High	0.150	0.089
Low to High	0.275	0.127
Low to Zero	0.050	0.063
High to Low	0.025	0.013
High to Zero	0.025	0.013
Low to Zero to High		0.013
Zero to Low to High		0.051
Zero to Low to Zero to Low		0.013

**Fig 2:**
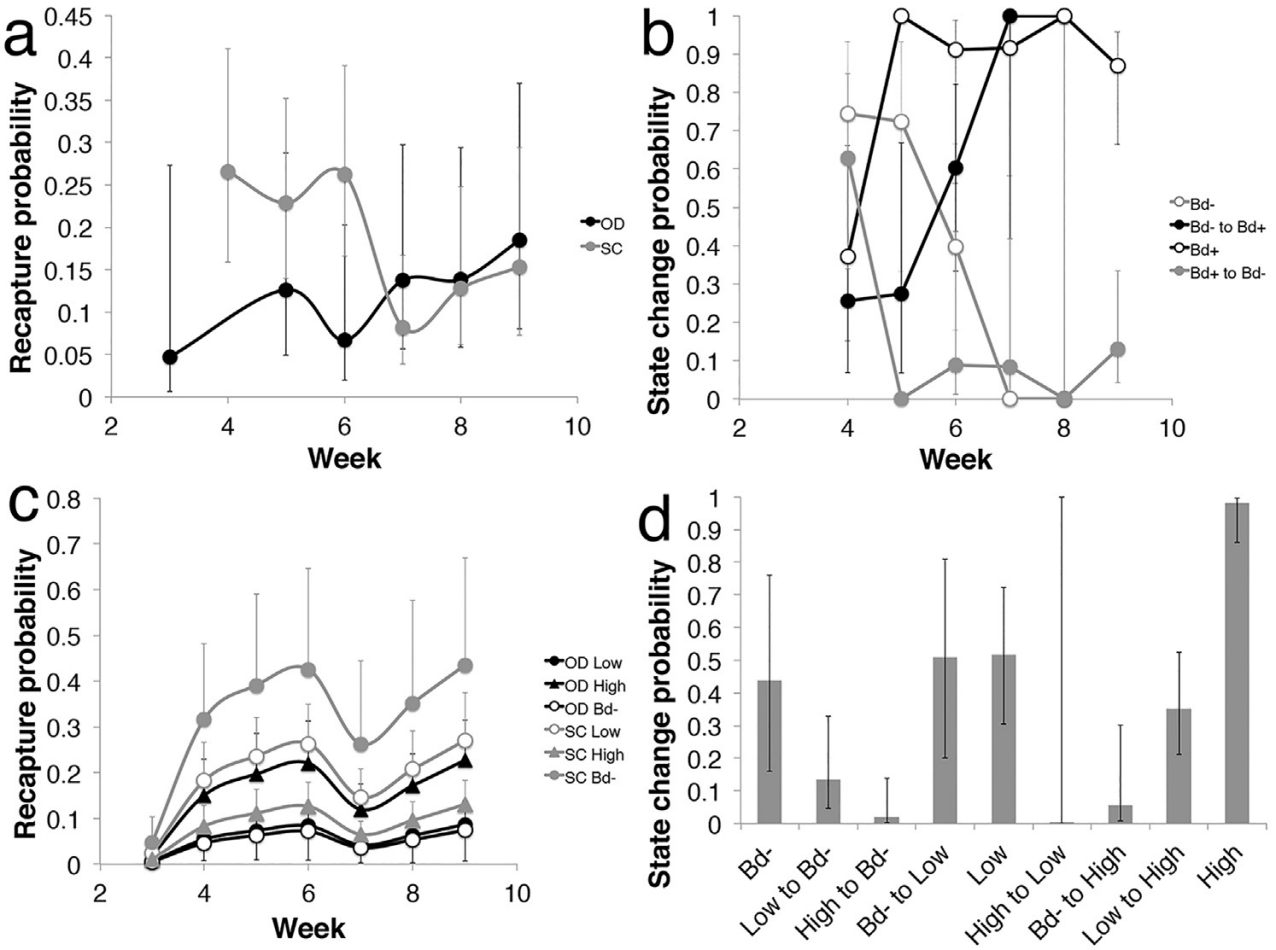
Recapture probability and State change probability. Conditional Arnason-Schwarz model in which outcome probabilities are (S) survival, (p) recapture, (Ψ) state change, and the variables that can influence the outcomes are (g) site, (t) time in weeks, (to) state at previous capture, (f) state at capture. Panels (a) and (b) represent the two-disease-state model, *Bd* positive (Bd+) and *Bd* negative (Bd-), in which the best model was S(g)p(g*t)Ψ(to*f*t). Panels (c) and (d) represent the three-disease-state model, *Bd* negative (Bd-), low infection intensity of >350ZE (Low) and high infection intensity of >350ZE (High), in which the best model was S(g*f)p(g*to+t)Ψ(to*f). (a) Recapture probability per week in a two-disease-state model. Factors included in the best model for recapture probability were site and week, Error bars indicate 95% confidence interval. (b) Probability of changing state per week in a two-disease-state model. Factors included in the best model for state change probability were week, infection state at current capture, and infection state at previous capture, and error bars indicate 95% confidence interval. (c) Recapture probability per week in a three-disease-state model. Factors included in the best model for recapture probability were site, state of infection and week. Error bars indicate standard error, and only one error bar included for figure clarity. (d) Probability of changing state in a three-disease-state model, error bars indicate 95% confidence interval. Sites are Oglives Dam (OD) and Sponar’s Creek (SC).

Similarly, infection intensity increased throughout the course of the breeding season (ANCOVA: F_11, 346_ = 6.216, p < 0.001) and differed between the two sites (ANCOVA: F_1,346_ = 6.002, p = 0.015), with Oglivies Dam having 2.02 times greater average infection intensity (d = 0.48) (6,698±16,522ZE) than Sponar’s Creek (3,315±10,034ZE) (Fig 2B).

Males had higher overall infection intensities than females (averaged across sites: males 5,497±736ZE; females, 2,730±2,513ZE) (ANCOVA: by sex, F_1,597_ = 612.369, p<0.01; by sex*week F_10,597_ = 2.335, p = 0.01), but infection intensity did not vary by site for each sex (ANCOVA: by sex*site, F_1,597_ = 1.289, p = 0.257).

Body condition was not affected by infection intensity (Linear regression ANOVA: F_1, 580_ = 0.62, p = 0.432). Male body condition did not vary between sites (ANCOVA: F_1,580_ = 0.812, p = 0.368), but did decrease by an average of 10.51% each week throughout the course of the breeding season (ANCOVA: F_11, 580_ = 4.984, p < 0.001; d = 0.1051). Body condition of the females did not vary between sites (ANCOVA: F_1, 36_ = 0.245, p = 0.626) or through time (ANCOVA: F_11, 36_ = 1.37, p = 0.26).

### CMR analysis

#### Two-disease-state model

Using a two state analysis (*Bd* positive and *Bd* negative) the two best fit models were Model 1) S(g)p(g*t)Ψ(to*f*t) (AIC = 927.706, deviance = 867.706, parameters = 30), and Model 2) S(.)p(g*t)Ψ(to*f*t) (AIC = 927.927, deviance = 869.972, parameters = 29) (Table 4). The outcomes explored were (S) survival probability, (p) recapture probability, and (Ψ) state change probability. The variables affecting each probability were (g) site, (t) time in weeks, (to) state of previous capture, (f) state of capture, and (.) indicates that no variable affected the probability. Neither model suggested that disease state influenced survival. In Model 1, survival probability per week differed between sites: animals at Oglivies Dam had a lower estimated survival probability per week (0.721, 95% CI, 0.555–0.843) than Sponar’s Creek (0.861, 95% CI, 0.726–0.935). In Model 2 survival probability was the same for all animals (0.821, 95% CI 0.722–0.890). Recapture and disease state change probability were equivalent in models 1 and 2. Recapture probability differed between sites and weeks but not for different disease states (Fig 2A). Recapture rate was lower earlier in the season for Oglivies Dam, while it was higher early in the season for Sponar’s Creek. Towards the end of the sampling period, both sites had similar weekly recapture rates. The probability of an animal changing infection state was dependent on time and disease status but not site (Fig 2B). Early in the season, there was a higher probability of staying *Bd* negative or clearing infection, but after week 7 the chance of staying *Bd* negative or clearing infection dropped to a very low probability. Early in the season animals were not likely to gain *Bd* infection or remain *Bd* positive. But after week 7 the likelihood of maintaining infection or becoming infected was high.

**Table 4:** AIC ranking for Conditional Arnason–Schwarz models. Model results for both the two-disease-state (*Bd* negative and *Bd* positive) and three-disease-state analysis (*Bd* negative, low *Bd* infection under 350ZE, and high *Bd* infection over 350ZE).

Model Variables*	Parameter	Deviance	AIC	Δ AIC
*2-State*				
S(g)p(g*t)Ψ(to*t)	30	867.706	927.706	0
S(.)p(g*t)Ψ(to*f*t)	29	869.972	927.972	0.266
S(g+f)p(g*t)Ψ(to*f*t)	31	867.697	929.697	1.991
S(f)p(g*t)Ψ(to*f*t)	30	869.971	929.971	2.265
S(g*f)p(g*t)Ψ(to*f*t)	32	867.377	931.377	3.671
S(f*g)p(g*t)Ψ(to*f*t)	32	867.377	931.377	3.671
S(g)p(g*t)Ψ(to*f)	18	900.469	936.469	8.763
S(g*f*to)p(g*to*t)Ψ(g*to*f*t)	67	835.799	969.799	42.093
S(.)p(.)Ψ(.)	3	987.247	993.247	65.541
*3-State*				
S(g*f)p(g*to+t)Ψ(to*f)	24	979.337	1027.337	0
S(g*f)p(g*to+t)Ψ(f)	21	987.779	1029.776	2.439
S(g*f)p(g*to+t)Ψ(to)	21	996.219	1038.219	10.882
S(g*f)p(g*to+t)Ψ(to*f*t)	60	921.865	1041.865	14.528
S(g)p(g*to+t)Ψ(to*f*t)	56	935.674	1047.674	20.337
S(g*f)p(g*t)Ψ(to*f*t)	62	925.296	1049.296	21.959
S(f)p(g*to+t)Ψ(to*f*t)	57	935.729	1049.729	22.392
S(g*f*t)p(g*to*t)Ψ(g*to*f*t)	113	859.464	1085.464	58.127
S(.)p(.)Ψ(.)	3	1079.595	1085.595	58.258

*Probabilities determined are (S) survival probability, (p) recapture probability, (Ψ) state change probability; and the variables that influence S, p and Ψ are (g) site, (t) time in weeks, (to) state of previous capture, (f) state of capture and no variable (.)

#### Three-disease-state model

When including three-disease-states in the CMR analysis (*Bd* negative, “low infection” with intensity of infection under 350ZE, and “high infection” with intensity of infection above 350ZE), the best fit model was S(g*f)p(g*to+t)Ψ(to*f) (AIC = 1027.424, deviance = 979.424, parameters = 26) (Table 4). Disease status and site were important factors influencing weekly survival. Uninfected animals from both sites had similar survival probabilities per week (Oglivies Dam: 0.766, 95% CI, 0.273–0.966; Sponar’s Creek: 0.760, 95% CI 0.552–0.891), and when infected with low *Bd* loads (Oglivies Dam: 0.994, 95% CI, 0.003–1.0; Sponar’s Creek: 0.997, 95% CI, 0.001–1.0). In contrast, when carrying a high *Bd* infection, weekly survival at Oglivies Dam was lower (0.460, 95% CI, 0.232–0.706) than survival at Sponar’s Creek (0.771, 95% CI, 0.423–0.939). Recapture probability was dependent on week, site, and state at previous capture (Fig 2C). Recapture probability was higher overall at Sponar’s Creek than at Oglivies Dam, but infection intensity impacted recapture rates differently at the two different sites. At Oglivies Dam, animals were more likely to be captured if they had high *Bd* infection intensity; but at Sponar’s Creek, *Bd* negative animals had the highest recapture probability. Infection state change probability was dependent on state of infection at current capture, and state of infection at previous capture, but not time or site (Fig 2D). While some animals remained *Bd* negative over the breeding season, most became infected. Once lightly infected, some animals appeared to clear the infection; however, if the animals showed a high intensity of infection, they were less likely to become *Bd* negative at the next capture.

### Reservoir hosts

A total of 94 *C*. *destructor* crayfish were sampled and all tested negative for *Bd* (Fig 1A). A total of 93 *C*. *signifera* individuals were tested for *Bd*. Infection prevalence was 100% (Fig 1A), and infection intensity was consistently high (3875±6691ZE) throughout the breeding season (Fig 1B).

## Discussion

### Density-dependence

Our study supports the concept of density-dependence of *Bd* infection because the 90–fold higher density site, Oglivies Dam, had 3.49 and 2.02 times higher prevalence and intensity, respectively, throughout the breeding season than the Sponar’s Creek site. This pattern has also been shown in studies of *Rana muscosa*, in which *Bd* infection also increased with increased population density [5,6,32]. The transmission and spread of *Bd* is known to have density-dependent disease transmission characteristics in other studies [4–6]. At higher population densities, uninfected animals are more likely to come in contact with infected animals and the pathogen in the environment, leading to an increase in direct and indirect transmission [33].

### Disease dynamics

Infection intensity and prevalence at the beginning of the season were lower at both sites, and then it increased until the end of the breeding season (Fig 1). The increase in infection intensity and prevalence is consistent with what has been observed for other aggregate breeders [13,34]. However, some *L*. *v*. *alpina* animals entered the breeding season with an infection suggesting that they are becoming infected outside the breeding season. These infected animals might have overwintered with infection after exposure to infection in the previous season; or, more likely, they gained infection before the breeding season while they were dispersed in terrestrial habitats. The abundance of terrestrial reservoirs could be a key factor determining exposure in the non-breeding season (see Reservoir Hosts section below). However, overwintering ecology and infection susceptibility are unknown in this species [23].

### CMR analysis

Recapture rates differed between the two sites. This was expected because they differed significantly in population density [30,35,36]. Animals in the smaller population, Sponar’s Creek, were more likely to be recaptured as recapture effort was relatively greater per individual (Table 2). Uninfected animals were more likely to be recaptured than infected animals at Sponar’s Creek (Fig 2C), which is a pattern observed in other frog species with *Bd* [14,15]. However, recapture probabilities were different at Oglivies Dam, where heavily infected animals were more likely to be recaptured. This is consistent with CMR analysis of other wildlife diseases [28]. While the heavily infected category for this study was >350ZE, animals likely do not become ill until a much higher infection load develops [37]. Oglivies Dam animals had higher infection intensities, and more animals were likely experiencing clinical signs of chytridiomycosis in the heavily infected category compared with animals at Sponar’s Creek. Heavily infected animals might be more easily recaptured because they are more lethargic. Therefore, if there is large heterogeneity in infection burden, it is important to include infection intensity in analyses to help tease apart its effects on recapture rates.

The results from the infection state change analysis suggest that the *Bd* transmission and recovery dynamics were similar in both populations. The two-disease-state analysis suggested that early in the season, some animals were able to recover from infection, but as the breeding season progressed, animals were less likely to recover and unlikely to remain *Bd* negative. The higher potential for recovery early on might occur because there were fewer heavily infected animals early in the season, and therefore, lower transmission rates and exposure and re-exposure to the pathogen would occur. When the recapture analysis was expanded to include three disease states, it showed that animals were very unlikely to recover from high infection intensity as seen by the high proportion of animals remaining at high infection intensity (Fig 2D). In contrast, recovery from low infection is more likely.

Results of analysis of factors influencing survival depended on whether infection intensity was included. In the two-disease-state model without considering infection intensity, the best-fit model suggested that disease state does not influence survival; however, the lower weekly survival probability for Oglivies Dam is confounded by the higher proportion of infected individuals with higher infection intensities as demonstrated by the three-disease-state model. In the three-disease-state model, survival of the highly infected animals differed between the sites, with Oglivies Dam animals having lower survival (0.46 weekly survival) than the highly infected animals at Sponar’s Creek, and the low infected and uninfected animals at both sites had similar weekly survival rates (0.76–0.99). It is possible that the site differences in survival suggested by the model were not due to site characteristics, but rather they represented differences in infection intensity between the sites that our model was unable to discern using the high intensity threshold of 350ZE. The lower survival of heavily infected animals at Oglivies Dam might be because Oglivies Dam animals were more heavily infected than Sponar’s Creek animals. These results support previous reports of the progression of *Bd* infection in susceptible species. Infection intensity builds over several weeks, and animals succumb to chytridiomycosis and die when heavy infection burdens are reached [6,13,38]. Analysing field data using multistate models is important to characterize disease dynamics in affected populations [39,40].

### Differences in the sexes

A limitation of this study is that females were never recaptured and so we were unable to determine the effect of disease on their behaviour and survival. However, for *L*. *v*. *alpina*, males are likely to be the drivers of *Bd* within the system due to their breeding behaviour. Males tend to be present in the breeding ponds for weeks at a time, whereas females arrive to mate and deposit their eggs and then return to their non-breeding habitat. Males have higher infection intensity throughout the breeding season because they spend more time in the breeding habitat increasing their chance of pathogen exposure, either through direct contact with other animals, or through indirect transmission in the aquatic environment. While these results suggest that females have lower levels of infection in the breeding pond during capture than males, female *L*. *v*. *alpina* have similar low year-to-year survivorship similar to males [19]. This suggests that total infection rates and disease outcomes are similar in both sexes.

### Reservoir hosts

Reservoir species likely play an important role in infection dynamics of *L*. *v*. *alpina*. Infected *L*. *v*. *alpina* die after the breeding season resulting in almost complete population turnover every year [19]. Tadpoles and juveniles leave the pond uninfected [19] and most first time breeders enter a pond uninfected. Under such circumstances, reservoir hosts are the likely cause of pathogen persistence. Two potential reservoir species were analysed in this study: an invasive crayfish species, *C*. *destructor*, and a sympatric frog species, *C*. *signifera*. We did not find crayfish carrying *Bd* infection, suggesting that *C*. *destructor* is not a reservoir species in the Australian Alps. However, we found 100% prevalence and high intensity of *Bd* infection in *C*. *signifera*. Intensity of infection in *C*. *signifera* is higher than *L*. *v*. *alpina* throughout the breeding season (Fig 1B). Additionally *C*. *signifera* do not vary in infection prevalence or intensity throughout the season suggesting that this species is tolerant of infection and is likely to be contributing to persistence and spread of this deadly pathogen similar to other reservoir hosts of this pathogen [41]. *Litoria v*. *alpina* can be found in close contact with *C*. *signifera* during the breeding season and in shared hibernation places during the non-breeding season. It is likely that infection in *L*. *v*. *alpina* is perpetuated by direct and indirect transmission of *Bd* from *C*. *signifera*.

### Management implications

Recent laboratory studies suggest that *L*. *v*. *alpina* might be evolving an immune response to fight infection [37], and selection for resistance is a possibility. However, artificial selection for disease resistance to chytridiomycosis has not been successfully attempted, and it is costly in terms of the research required [42,43]. In addition, at higher density site the lower survivorship when heavily infected might reduce the opportunity for the evolution of resistance mechanisms that could permeate the population. Therefore, alternative management strategies might be more efficient in the short term to help secure the conservation of species [42]. Our study supports the density-dependence of *Bd*. Our site with higher population density of *L*. *v*. *alpina* had higher infection prevalence and intensity and lower survival for heavily infected frogs. In order to promote survivorship and decrease effects of disease, management strategies might aim to decrease population densities. Such management techniques might include increasing the area of the water body while maintaining the same size of suitable breeding habitat within the pond or decreasing environmental disease transmission by increasing water replacement using increased flow into and out of the ponds that *L*. *v*. *alpina* inhabit.

*Litoria*. *v*. *alpina* are highly susceptible to *Bd* infection, rarely recover from infection, and have near complete population turnover each year in populations with endemic *Bd* infections [19]. Therefore, high recruitment enables some populations to persist despite the impact of chytridiomycosis, and is crucial to the persistence of *L*. *v*. *alpina* [15,19,44]. While reproductive output in persisting populations is sufficient to maintain populations, it is a risky mechanism for population persistence. Drought is a high risk factor for decreased progeny survival as evidenced by *L*. *v*. *alpina* having been extirpated from all ephemeral water bodies [23]. Management strategies to ensure consistent annual survival of progeny by reducing the effects of drought by increasing water body size and water input would promote population persistence [23]. Increasing water body size and water input (while maintaining breeding habitat size) might serve to both promote lower population densities and enable consistent recruitment. This strategy might be effective in sustaining some reintroduced populations. Because *C*. *signifera* appears to be maintaining infection within the system, reducing their abundance or reintroducing *L*. *v*. *alpina* into sites where they are absent could promote *L*. *v*. *alpina* conservation.

## Supporting Information

The following supporting information is available for this article online.

S1 Fig Map of Kosciuszko National Park, New South Wales, Australia study sites. Oglivies Dam 35° 57' 29" S, 148° 24' 4" E: Elevation 1382m. Sponar’s Creek, 36° 21' 32.4" S, 148° 30' 0" E: Elevation 1515m. Kiandra, 35° 52' 1" S, 148° 29' 53" E: Elevation 1358m, where the crayfish were collected. The white lines indicate state lines, Victoria to the West and Australian Capital Territory to the Northeast. The grey lines indicate major roadways. Scale bar = 10km. Map data reprinted from Google Imagery under CC BY license, with permission from TerraMetrics, original copyright 2015.
(TIF)

S2 Fig Air and water temperatures in degrees Celsius at the sites. Sites are Oglivies Dam and Sponar’s Creek. Error bars are standard error. Temperatures were collected with iButtons places at the sites.
(TIF)

S1 File Supplemental Methods. Further information on the study species, study sites, weather data collection, and individual marking methods.
(DOCX)

S1 Table Collection data of *L*. *v*. *alpina*. Data includes site, sex, date of collection, SVL, mass, and infection load or each animal throughout the study.
(XLSX)

S2 Table Infection status of potential reservoir hosts. Species, date of collection, site and infection status and intensity.
(XLSX)
