# Supplementary figures and images for: Dynamics of Chytridiomycosis during the Breeding Season in an Australian Alpine Amphibian
Source: PLoS One. 2015 Dec 2;10(12):e0143629. doi: 10.1371/journal.pone.0143629 (PMC4668081; doi:10.1371/journal.pone.0143629)

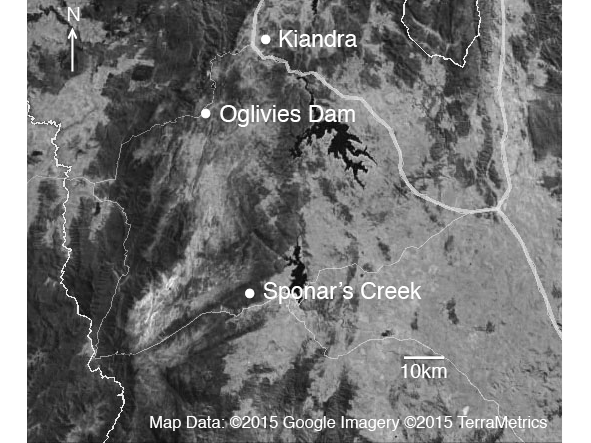

Supplement: S1 Fig — Oglivies Dam 35° 57' 29" S, 148° 24' 4" E: Elevation 1382m. Sponar’s Creek, 36° 21' 32.4" S, 148° 30' 0" E: Elevation 1515m. Kiandra, 35° 52' 1" S, 148° 29' 53" E: Elevation 1358m, where the crayfish were collected. The white lines indicate state lines, Victoria to the West and Australian Capital Territory to the Northeast. The grey lines indicate major roadways. Scale bar = 10km. Map data reprinted from Google Imagery under CC BY license, with permission from TerraMetrics, original copyright 2015. (TIF) [file pone.0143629.s001.tif]

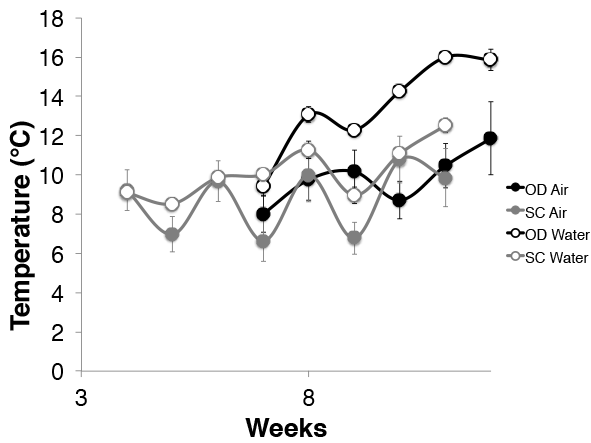

Supplement: S2 Fig — Sites are Oglivies Dam and Sponar’s Creek. Error bars are standard error. Temperatures were collected with iButtons places at the sites. (TIF) [file pone.0143629.s002.tif]
