# Supplementary material for: Dynamics of Chytridiomycosis during the Breeding Season in an Australian Alpine Amphibian
Source: PLoS One. 2015 Dec 2;10(12):e0143629. doi: 10.1371/journal.pone.0143629 (PMC4668081; doi:10.1371/journal.pone.0143629)
Supplement: S1 File — Further information on the study species, study sites, weather data collection, and individual marking methods. (DOCX) [file pone.0143629.s003.docx]

**Supplemental Methods**

**Study Species**

*Litoria verreauxii alpina* occurs in sub-alpine woodland, grasslands, and bog environments. Breeding populations occur in streamside pools and all current populations occur in or near permanent water bodies; many of which are artificial, such as small dams and reservoirs [1,2]. Historically the species was also common in ephemeral wetland habitats but appears to have been extirpated from these areas [1,2]. Calling occurs from late winter to early summer [3]. Eggs are laid in pools around submerged vegetation in large jelly-like clumps; larvae hatch within a few days [3]. Tadpoles have been recorded from November to January and metamorphose from December to January [4]. During the non-breeding season, individuals disperse from the breeding habitat and hide amongst leaf litter, logs, or stones [3]. *Litoria. v. alpina* is highly susceptible to *Bd* in the lab and succumb to infection in just three weeks [5].

**Study Sites**

Oglivies Dam occurs at 1383 elevation and consists of two small ponds with an ephemeral creek feeding and draining. The combined water bodies occupy 0.17 hectares, and are up to 1.5m deep. The site is 20m from a seasonal road open to the public from December to May. Animals are found throughout the entire water bodies. Sponar’s Creek occurs at 1515m elevation and is a large pond 5m from a main road and 50m from a hotel. The pond occupies 1.8 hectares and is fed and drained by a permanent creek. Suitable breeding habitat for *L. v. alpina* is confined to the edges of the pond, which is where animals were captured. Both sites are permanent water bodies. Study site area was measured as the water body plus a 1 m perimeter where the animals come to breed.

**Weather data**

Mean weekly air temperatures were similar for both sites but mean weekly water temperature were higher and with a steady increase over time at Oglivies Dam due to the smaller size of the pond (S. Fig. 2). Both sites temperatures were below the range for optimum *Bd* growth, which is between 13°C and 25°C [6].

**Individual identification**

Upon first capture a toe tip (right fourth digit) was collected and stored in 98% ethanol for a genetic sample and a marker of recapture. Inflammation at site of tipping was never observed upon recapture. Animals were individually identified by photographs of their dorsum and left side. Left side photographs were used most often for identification, and dorsum photographs were used as verification when needed. *Litoria v. alpina* are variable in colouration and pattern, but as the study was short (10 weeks at each site), pattern change was not a concern. Photographs were chosen as an identification method because a laboratory study conducted on this species demonstrated that neither passive integrative transponder (PIT) tags nor visual implant elastomer (VIE) tags were reliable tagging methods, and toe clipping, while reliable, was slow to heal and caused infection in some individuals in the laboratory [7].

**References**

1. Osborne W, Hunter D, Hollis G (1999) Population declines and range contraction in Australian alpine frogs. In: Campbell A, editor. Declines and Disappearances of Australian Frogs. Canberra, ACT: Biodiversity Group Environmental Australia, Vol. 1. pp. 145–159.

2. Scheele BC, Hunter DA, Skerratt LF, Brannelly LA, Driscoll DA (2015) Low impact of chytridiomycosis on frog recruitment enables persistence in refuges despite high adult mortality. Biol Conserv 182: 36–43.

3. Gillespie GR, Osborne WS, McElhinney NA (1995) The conservation status of frogs in the Australian Alps: a review. Report to Australian Alps Liaison Committee, Canberra, ACT, Australia.

4. Hunter DA, Osborne WS, Smith MJ (1998) Distribution and abundance of the alpine tree frog (*Litoria verreauxii alpina*) in the Australian Alps National Parks. Report on the first seasons survey (1996-1997). Report to NSW NPWS.

5. Bataille A, Cashins SD, Grogan L, Skerratt LF, Hunter D, McFadden M, et al. (2015) Susceptibility of amphibians to chytridiomycosis is associated with MHC class II conformation. Proceeding R Soc B 282: 20143127.

6. Stevenson LA, Alford RA, Bell SC, Roznik EA, Berger L, Pike DA (2013) Variation in thermal performance of a widespread pathogen, the amphibian chytrid fungus *Batrachochytrium dendrobatidis*. PLoS One 8: e73830.

7. Brannelly LA, Berger L, Skerratt LF (2014) Comparison of three widely used marking techniques on adult anuran species *Litoria verreauxii alpina*. Herpetol Conserv Biol 9: 428–435.
